# Subcortical Dopamine and Cognition in Schizophrenia: Looking Beyond Psychosis in Preclinical Models

**DOI:** 10.3389/fnins.2020.00542

**Published:** 2020-06-03

**Authors:** Kyna-Anne Conn, Thomas H. J. Burne, James P. Kesby

**Affiliations:** ^1^Queensland Brain Institute, The University of Queensland, St Lucia, QLD, Australia; ^2^Queensland Centre for Mental Health Research, Wacol, QLD, Australia; ^3^QIMR Berghofer Medical Research Institute, Herston, QLD, Australia

**Keywords:** operant tasks, goal-directed behavior, reversal learning, rodent, translation

## Abstract

Schizophrenia is characterized by positive, negative and cognitive symptoms. All current antipsychotic treatments feature dopamine-receptor antagonism that is relatively effective at addressing the psychotic (positive) symptoms of schizophrenia. However, there is no clear evidence that these medications improve the negative or cognitive symptoms, which are the greatest predictors of functional outcomes. One of the most robust pathophysiological observations in patients with schizophrenia is increased subcortical dopamine neurotransmission, primarily in the associative striatum. This brain area has an important role in a range of cognitive processes. Dopamine is also known to play a major part in regulating a number of cognitive functions impaired in schizophrenia but much of this research has been focused on cortical dopamine. Emerging research highlights the strong influence subcortical dopamine has on a range of cognitive domains, including attention, reward learning, goal-directed action and decision-making. Nonetheless, the precise role of the associative striatum in the cognitive impairments observed in schizophrenia remains poorly understood, presenting an opportunity to revisit its contribution to schizophrenia. Without a better understanding of the mechanisms underlying cognitive dysfunction, treatment development remains at a standstill. For this reason, improved preclinical animal models are needed if we are to understand the complex relationship between subcortical dopamine and cognition. A range of new techniques are facillitating the discrete manipulation of dopaminergic neurotransmission and measurements of cognitive performance, which can be investigated using a variety of sensitive translatable tasks. This has the potential to aid the successful incorporation of recent clinical research to address the lack of treatment strategies for cognitive symptoms in schizophrenia. This review will give an overview on the current state of research focused on subcortical dopamine and cognition in the context of schizophrenia research. We also discuss future strategies and approaches aimed at improving the translational outcomes for the treatment of cognitive deficits in schizophrenia.

## Introduction

The dopaminergic system is thought to be involved in both the etiology of schizophrenia and the regulation of a number of cognitive domains. Examination of the relationship between dopamine and cognition has largely focused on the role of cortical dopamine because the prefrontal cortex (PFC) in particular, is known to regulate a number of executive functions (Braver and Cohen, 1999; Orellana and Slachevsky, 2013). The role of subcortical dopamine systems and cognition in schizophrenia has received less attention. This is a consequence of the fact that the therapeutic action of all antipsychotic medication features the blockade of dopamine transmission, based on a number of molecular imaging studies (Seeman and Lee, 1975; Creese et al., 1976; Richtand et al., 2007; Howes et al., 2009a; Miller, 2009), but seemingly fails to improve cognitive impairments (Swartz et al., 2008). In some cases, antipsychotics may even exacerbate these deficits (Stip, 2006). While research into the pharmacodynamics of antipsychotic medication has advanced significantly, the relationship between dopamine and cognition is an important avenue to explore considering its potential influence on functional outcomes.

Currently, the overall consensus is that antipsychotic treatments seemingly have little to no effect on improving the cognitive symptoms, observed with both first- and second-generation antipsychotic medications (Hill et al., 2010; Frazier et al., 2012). Previously, a number of studies attempted to delineate the effects of both types of antipsychotics, with most suggesting second-generation antipsychotic administration had a more marked improvement in cognitive functioning (Lee et al., 1994; Meltzer and McGurk, 1999; Meltzer and Sumiyoshi, 2003; Sumiyoshi et al., 2013). While these studies reported significant improvements in cognition, the results were domain-specific and were confounded by issues such as duration of treatment and practice effects (Keefe et al., 2007). Other major inadequencies highlighted in these studies included poor experimental design, lack of appropriate control groups, insufficient washout periods, use of several medications and failure to account for dosage or duration of administration. It is also important to note that second-generation antipsychotics can induce serious metabolic side effects such as obesity and type II diabetes, illnesses that are strongly linked with cognitive impairments on their own (MacKenzie et al., 2018).

While most studies focus on cortical dopamine and cognition, subcortical regions such as the basal ganglia (a group of nuclei responsible for the coordination of a variety of motor functions) also have a primary role in complex cognitive processing (Middleton and Strick, 2000). Recent clinical evidence indicates that alterations in dopaminergic function in schizophrenia are primarily driven by changes in the associative striatum (Laruelle et al., 2005; Howes et al., 2009b; Kegeles et al., 2010). The associative striatum is heavily involved in a range of cognitive and decision-making processes and is anatomically defined as being part of the medial caudate and ventral putamen (Kesby et al., 2018). This suggests that understanding the role of subcortical dopamine in the cognitive deficits observed in schizophrenia may provide a better understanding of cognition in general, and identify novel approaches to treating these complex symptoms.

Cognitive dysfunction is thought to be one of the greatest predictors of functional outcomes in patients (Green et al., 2004). Impairments are observed in those at ultra-high-risk and with first-episode psychosis, as well as first-order relatives (Keshavan et al., 2010; Morales-Munoz et al., 2017; Lam et al., 2018). As cognitive symptoms present before the prodromal period and persist throughout the development of schizophrenia, cognitive impairment could be a biomarker for at-risk patients and a target for early prevention (Heinrichs and Zakzanis, 1998). Given the role of the associative striatum in decision-making processes, understanding the effects of altered dopamine function in this region on cognitive function is essential. For example, the associative striatum is engaged during two different components of decision-making, goal-directed action and reversal learning, both of which are impaired in schizophrenia (Redgrave et al., 2010; Morris et al., 2015). In this review, we will address the role of subcortical dopamine in the decision-making deficits observed in schizophrenia and discuss the evidence from preclinical studies which have sought to identify the underlying neural circuitry. We believe that a new approach is necessary to develop novel therapeutic targets to treat the cognitive symptoms of the disorder. To reduce the current translational gap between basic and clinical research, we suggest a shift in focus from categorical clinical measures to experimental psychopathology, i.e., elucidating the mechanisms that contribute to the etiology, exacerbation or maintenance of abnormal behavior (Forsyth and Zvolensky, 2001). With advances in genetic tools for use in animal models, manipulations of the neural circuitry and measurement of the consequent effects on cognition will also provide an avenue to improve translational outcomes.

## Subcortical Dopamine Abnormalities in Schizophrenia

Dopamine regulates a range of motor, limbic and cognitive functions. Based on evidence from a number of disorders (e.g., Parkinson’s disease, attention deficit hyperactivity disorder, obsessive-compulsive disorder and schizophrenia), dysfunction of the dopamine system is thought to contribute to a range of neuropsychiatric symptoms. Dopamine neurons are located primarily in the midbrain, specifically in the substantia nigra and ventral tegmental area. Dopaminergic projections from the midbrain are divided into the mesocortical and mesolimbic systems (dopamine cells that arise in the ventral tegmental area and project to the PFC and limbic striatum, respectively), and the nigrostriatal system (dopamine cells that arise in the substantia nigra and project to the associative striatum). The associative striatum also receives rich connections from cortical areas including the dorsolateral PFC, anterior cingulate cortex (ACC), and orbitofrontal cortex (OFC), and has reciprocal thalamic connectivity (Haber, 2016). It is the associative striatum’s role in gating incoming cortical input that makes it fundamental in maintaining the ability to adapt our choices to environmental changes (i.e., decision-making; Sharpe et al., 2018).

Alterations in dopamine neurotransmission have long been associated with the pathophysiology of schizophrenia. Early perturbations in the dopaminergic system were hypothesized to be a causative factor in the development of the disorder (Weinberger, 1987), driving both psychotic and cognitive symptoms (Laruelle et al., 2003). Recent evidence suggests that cortical dopamine function is decreased in schizophrenia (Slifstein et al., 2015), which may contribute to cognitive dysfunction. However, this does not preclude a role for subcortical dopamine systems. As such, this review will focus on subcortical dopamine systems and discuss cortical dopamine only when relevant to these cognitive processes (and to confirm when functional outcomes are insensitive to cortical dopamine changes).

In contrast with earlier hypotheses centered on mesolimbic dopamine (Laruelle et al., 2003), the current evidence supports a role for associative striatal dopamine dysfunction in schizophrenia. For example, a landmark study by Laruelle et al. (2005) demonstrated that the striatal localization of dopaminergic hyperfunction was primarily restricted to the associative, and not the limbic striatum. The results of this positron emission tomography (PET) imaging study challenged the widely accepted view that the therapeutic effects of antipsychotic drugs are derived from actions in the limbic striatum whereas actions in the associative striatum are responsible for the motoric side effects (Laruelle et al., 2005). It has subsequently been shown that dopaminergic hyperactivity is present before the onset of the disorder, is predominately found in the associative striatum, and increases in those who transition to schizophrenia (Howes et al., 2009b). Dopamine hyperactivity also correlates with the severity of symptoms, as well as cognitive dysfunction (Howes et al., 2009b). In addition, elevated dopamine synthesis capacity was seen in the midbrain origins of dopamine neurons as well as their striatal terminals, with this finding also being linked to symptom severity in the disorder (Howes et al., 2013). Together, these studies support the notion that subcortical dopamine dysfunction and, in particular, dopaminergic alterations in the associative striatum, may be the main impetus for multiple symptoms of schizophrenia.

## The Role of the Associative Striatum in Cognitive Dysfunction

Cognitive dysfunction in schizophrenia spans a range of domains, including working memory, verbal speed, attention and executive function, and greatly impacts on patients’ lives (Green et al., 2000; Fujii et al., 2004; Green et al., 2004). Widespread functional and structural changes are observed in most cortical areas in schizophrenia (Brugger and Howes, 2017; Li et al., 2017) and undoubtedly contribute to cognitive dysfunction. However, subcortical dopamine systems also play specific roles in regulating multiple aspects of cognitive performance. Therefore, cognitive deficits driven by alterations in subcortical dopamine systems are likely located in substructures that feature dense cortical connectivity (Nieoullon, 2002), such as the associative striatum.

A number of clinical research findings support the involvement of the associative striatum in the cognitive deficits observed in schizophrenia patients. For example, structural changes in the size of the associative striatum in those with schizophrenia correlate with performance in cognitive tasks assessing executive functions (Levitt et al., 2013). Decreased striatal dopamine synthesis capacity, in patients with symptomatic remission of positive symptoms, mediates a range of cognitive symptoms (Avram et al., 2019). Changes in associative striatal activation during goal-directed behavior have also been shown to underlie performance deficits in schizophrenia (Morris et al., 2015). These examples support the established understanding that the associative striatum contributes directly to decision-making, specifically in action selection and initiation, integrating sensorimotor, cognitive and motivational information (Balleine et al., 2007). These processes are critical for instrumental learning and the ability to adapt behavior in the face of changing information. When understanding the role of the associative striatum in cognition, we must also consider the complexity of subcortical dopamine signaling more generally. The mesolimbic dopamine system encodes signals that allow the prediction of reward outcomes and are thought to mediate reward-related adaptation and learning (Gradin et al., 2011; Hauser et al., 2017). Limbic dopamine therefore impacts autoshaping behavior as well as reward learning processes, such as probabilistic learning (Markou et al., 2013), and is thought to contribute to motivational and reward deficits in schizophrenia (Der-Avakian et al., 2016).

Multiple studies have observed the absence of a relationship between antipsychotic use and cognitive improvement in those with schizophrenia, suggesting that dopamine D_2_ receptor signaling does not account for these findings *per se*. However, it is known that blockade of D_2_ receptors in the striatum is a major factor in causing acute drug-induced extrapyramidal side effects (EPS). EPS can further complicate the relationship between antipsychotic medication and cognitive function (Meltzer et al., 1999). The extrapyramidal system, as used in anatomy, defines part of the motor system network (other parts of the motor cortex reach their targets via the pyramidal tract). Thus, symptoms of EPS include dystonia, akathisia, parkinsonism, bradykinesia, tremor, and tardive dyskinesia, and antipsychotic treatment is often discontinued due to these intolerable side effects. The main distinguishing features between first- and second-generation antipsychotics is that second-generation antipsychotics tend to have a more potent blockade of serotonin receptors (5HT-2A) and weak blockade of D_2_ receptors, which results in lower rates of EPS (Meltzer et al., 1999). So even though all efficacious antipsychotic medications target the aforementioned dopaminergic abnormality in the striatum, there is little evidence to support improvements in cognition (Miller, 2009).

Both the Clinical Antipsychotic Trials of Intervention Effectiveness (CATIE) and the European First Episode Schizophrenia Trial (EUFEST) failed to show any effectiveness of second-generation antipsychotics in the treatment of cognitive symptoms in schizophrenia (Keefe et al., 2007; Davidson et al., 2009). These trials encompassed a large sample size with features reflective of the general schizophrenia population, showing that antipsychotic drugs are very similar in their action across chemical classes with these similarities extending to their effects on cognition. Higher lifetime dose-years were significantly associated with poorer cognitive performance and the effects of first- and second-generation antipsychotics did not differ (Husa et al., 2017). So, the superiority of second-generation antipsychotics was also called into question during these trials, with mixed results (Desamericq et al., 2014; Nielsen et al., 2015). Most importantly, the effect size for any cognitive improvement observed in these trials was small with spurious clinical significance (Heinrichs, 2007; Keefe et al., 2007).

Furthermore, to add to the complexity of understanding this relationship, there is some evidence suggesting that antipsychotic medication may worsen cognitive dysfunction. The therapeutic effects of these medications are known to treat the psychotic symptoms via a blockade of the D_2_ receptors and a study that stemmed from the CATIE trials attempted to elucidate the effects that this blockade had on neurocognitive performance (Creese et al., 1976). By evaluating the impact of estimated D_2_ receptor occupancy with antipsychotic drugs on cognitive performance, they were able to show that depending on the level of occupancy, these medications may increase the risk of EPS and also increase the chance of worsening cognitive impairment (Sakurai et al., 2013). This has been shown to impact on specific cognitive domains as well, for example, excessive D_2_ receptor occupancy correlates with attention deficit in late-life schizophrenia and a decrease in working memory performance (Uchida et al., 2009; Kim et al., 2013). Furthermore, in first episode psychosis patients, neuropsychological impairments are seemingly related to the pharmacodynamics and antipsychotic medication dosing regimens, specifically for verbal memory and motor function (Baitz et al., 2012).

Other effects of current antipsychotic treatments include alterations in functional connectivity in patients with long-term use (Bolding et al., 2012). This can be problematic when dysconnectivity in schizophrenia is considered to be a phenotype that may be due to either degenerative, developmental or genetic mechanisms (Meyer-Lindenberg and Weinberger, 2006). Another possible reason for the inefficacy of antipsychotic medication not alleviating cognitive symptoms is the potential role of the D_1_ receptor system, and not the D_2_ receptor system, contributing to cognitive dysfunction. It has been shown in a PET imaging study that binding of radioligand to D_1_ receptors was reduced in the PFC of drug-free patients with schizophrenia in comparison to healthy controls, and this correlated with severity of cognitive symptoms and performance on a set shifting task measuring cognitive flexibility (Okubo et al., 1997).

Seemingly, most research on cognition in schizophrenia has focused on executive functions. This may be problematic considering that executive functions include any process that relies on the PFC. The importance of cortico-striatal circuits, and the associative striatum in particular, suggests that the prevailing presumption that the PFC is the sole contributor to deficits in executive function, may have overlooked an important avenue for better understanding these deficits. Since it is clear that dopamine plays a role in both cognition and the therapeutic action of current drugs, it is important to understand how dopamine alterations in the brain may lead to cognitive dysfunction. The recent evidence supporting subcortical dopamine’s definitive role in the pathogenesis of the disorder may be key to predicting outcomes and responses to antipsychotic treatment (Kaar et al., 2019).

### The Functional Neuroanatomy of the Striatum

The striatum is involved in the coordination of multiple aspects of cognition, including motor- and action-planning, decision-making, motivation, reinforcement and reward perception (Balleine et al., 2007). However, the striatum can be parcellated into functional subregions which include the aforementioned associative and limbic, as well as the sensorimotor striatum (Heilbronner et al., 2016; Kesby et al., 2018). In rodents, these approximately correlate anatomically with the dorsomedial, ventral and dorsolateral striatum, respectively (see Table 1 for more detailed anatomical descriptions). In this current review, we will primarily use the functional names (i.e., associative, sensorimotor and limbic), and in the case of experimental manipulations, classified only by their neuroanatomical description (dorsomedial etc.), we will include the equivalent functional nomenclature in parenthesis. Each functional division of the striatum has a differing role in features of cognitive and reward processing. The associative learning of stimuli (i.e., formation of action-outcome associations) and action selection between competing alternatives is dependent on associative striatal function. The process of habit formation is thought to be dependent on activity in the sensorimotor striatum, whereas the motivational modulation of motor behavior is dependent on the limbic striatum (Liljeholm and O’Doherty, 2012). Generalized hypotheses of information flow during decision-making processes suggest that the limbic striatum encodes motivational variables, which are used by cortical subregions and the associative striatum for action selection and implementation. After sufficient training/repetition, this information is encoded by the sensorimotor striatum into a habit-based response (Pessiglione et al., 2007; Schmidt et al., 2012).

**TABLE 1:** Comparative striatal functional and neuroanatomical nomenclature.

**FUNCTIONAL REGION**	**HUMAN**	**RODENT**
**ASSOCIATIVE**	Medial caudate Ventral putamen	Dorsomedial striatum/caudate putamen
**SENSORIMOTOR**	Dorsolateral caudate Dorsolateral putamen	Dorsolateral striatum/caudate putamen
**LIMBIC**	Ventral striatum Nucleus accumbens	Ventral striatum Nucleus accumbens

The associative striatum plays an important role in instrumental learning, whereby reinforcement or punishment is used to increase or decrease the probability that a behavior will occur again in the future (Hall, 2002; Day et al., 2007). Instrumental learning can be goal-directed, which is a highly adaptive form of learning that requires the recruitment and integration of information from higher cortical regions such as the PFC, ACC and OFC. Essentially, the associative striatum accumulates this information to direct action-selection and decision-making (Yartsev et al., 2018). This is of relevance to schizophrenia, as it has been shown that corticostriatal control of goal-directed action is impaired. Specifically, those with schizophrenia are unable to integrate action-outcome learning to guide choice, a finding which has been shown to correlate with a reduction in associative striatal activity (Morris et al., 2015). The role of the limbic striatum is centered on motivational behavior, as evidenced by its involvement in the ability to predict the outcome of rewards (Schultz, 2000; Knutson et al., 2001; Tanaka et al., 2004). Not surprisingly, reduced activation in the ventral striatum has been correlated with the severity of negative symptoms in medication-free patients and in the response to cues predicting the outcome of rewards (Juckel et al., 2006; Nielsen et al., 2012). While research has predominantly focused on the role of the limbic striatum in the pathogenesis schizophrenia, little is known about the role of the associative striatum in the aberrant encoding of cortical decision-making processes observed in patients (Brunelin et al., 2013; Strauss et al., 2014).

## Reducing the Translational Gap With Improved Preclinical Tests

Although our knowledge of brain circuitry and schizophrenia neurobiology has advanced considerably in the past decade, drug development is at a standstill. Better translation between preclinical and clinical studies is necessary in order to identify novel treatment approaches (Pratt et al., 2012; Kesby et al., 2018). The lack of cognitive improvement in response to antipsychotic medication has led to a shift in research, focusing more on the development of drugs to improve cognition in those with schizophrenia (Floresco et al., 2005; Young and Geyer, 2015). Unfortunately, drugs that appear to improve performance in animal models often do not show the same positive effects in the clinical population (Castner et al., 2000; George et al., 2007). Consequently, a number of initiatives have been established to examine dimensions of human behavior (e.g., attention, reward learning, memory) in order to facilitate novel research approaches to understand how structure and function of the brain impact neuropsychiatric impairments (Marder and Fenton, 2004; Carter and Barch, 2007; Insel, 2014). Importantly, these approaches have led to the development of comparative preclinical cognitive protocols and recommendations to improve the translational capacity in schizophrenia research (Young et al., 2009; Moore et al., 2013; Nikiforuk, 2018).

The combined use of sensitive and highly translatable cognitive tasks in combination with manipulations of the brain, relevant to schizophrenia, will help to reduce the current translational gap (Carandini and Churchland, 2013; Kesby et al., 2015). A range of pharmacological and genetic tools are now available in preclinical research that will allow us to elucidate the brain regions and molecular mechanisms behind some of the cognitive deficits in schizophrenia. As the associative striatum is involved in goal-directed behavior and reversal learning, both of which are impaired in schizophrenia, understanding the ability to select actions that guide choices is integral to understanding the link between striatal dopamine, cognition and schizophrenia (Kesby et al., 2018; McCutcheon et al., 2019).

## Examining the Role of the Associative Striatum in Goal-Directed and Flexible Decision-Making

We have recently advocated a move in research focus to behavioral phenotypes that are consistent with the underlying neuroanatomical and biological features of schizophrenia (Kesby et al., 2018). Based on emerging evidence supporting the role of the associative striatum in this disorder, it is clear that the cognitive domains of associative learning, goal-directed action and reversal learning are key targets for further investigation, and will be the focus for the rest of this review. The rationale is that the striatum is heavily involved with the selection of a motor plan (goal-directed action) by integrating the relationship between outcomes and their relative values (associative learning), and is how an animal can make a choice or adapt its behavior (Cox and Witten, 2019). These processes are encompassed under the umbrella of “decision-making,” a core but complex part of daily functioning that requires the use of higher-order cortical areas and subcortical brain structures such as the striatum (Goulet-Kennedy et al., 2016).

In terms of circuitry, the striatum is situated within multiple cortico-subcortical loops, receiving input from the cortex and thalamus, with reciprocal outputs to the cortex via the thalamus, making striatal function an integral part of decision-making (Redgrave et al., 2010). A number of cognitive processes are required to make a decision, including perception, attention, working memory, associative learning, long-term memory, adaptation and planning, before a choice or action selection is made (Young and Geyer, 2015). There are also a variety of tasks that are dependent on subcortical regions, with these mainly relating to decision-making based on action-outcome learning and reward feedback (Carandini and Churchland, 2013). It should be noted that associative learning is an integral component of both goal-directed action and reversal learning. By focusing on the aforementioned cognitive processes, we may be able to reveal behavioral responses that are consistent with the altered pathophysiological features of schizophrenia.

### Goal-Directed Behavior in Schizophrenia

Goal-directed behavior is wide ranging and allows us to understand the complex process of decision-making. The main associative account of goal-directed action is a response-outcome account that begins with the consideration of possible response alternatives and is followed by the evaluation of their consequences. This is underpinned by the formation of action-outcome contingencies via associative learning processes and has been extensively examined in rodents and humans alike (Friedel et al., 2014). A number of studies have proposed models for how goal-directed behavior is impacted in schizophrenia (Frith, 2000). One model in particular suggests that negative symptoms are associated with a deficit in action initiation and positive symptoms are associated with deficits in cognitive control, with disorganized symptoms associated with deficits in contextual information integration (Rinaldi and Lefebvre, 2016). In a study investigating goal-directed planning and action in a virtual environment, impairments in these processes were observed in those with schizophrenia (Siddiqui et al., 2019). In the context of a simulated everyday errands task, people with schizophrenia exhibited both a reduced capacity and efficiency to complete the task, indicating that goal-directed behavioral impairments can manifest as diminished real-world motivational and functional behavior. Understanding the interaction between schizophrenia pathophysiology and goal-directed behavior may therefore be essential for improving functional outcomes in patients.

Imaging studies in human participants have helped to establish the brain areas and circuits that mediate goal-directed behavior. For example, enhanced medial PFC and posterior cingulate cortex activity has been observed during action selection in the training phase of a goal-directed behavioral task (Eryilmaz et al., 2017). In the same study, early phases of associative learning, i.e., goal-directed learning, were associated with increased activation in the frontoparietal control network (which serves to instantiate new task states by flexibly interacting with other control networks) and the caudate (which encompasses most of the associative striatum). In contrast, late phase learning, i.e., habit formation, showed activation of default mode regions that are more active during times of rest as opposed to times of cognitive activity.

**FIGURE 1:**
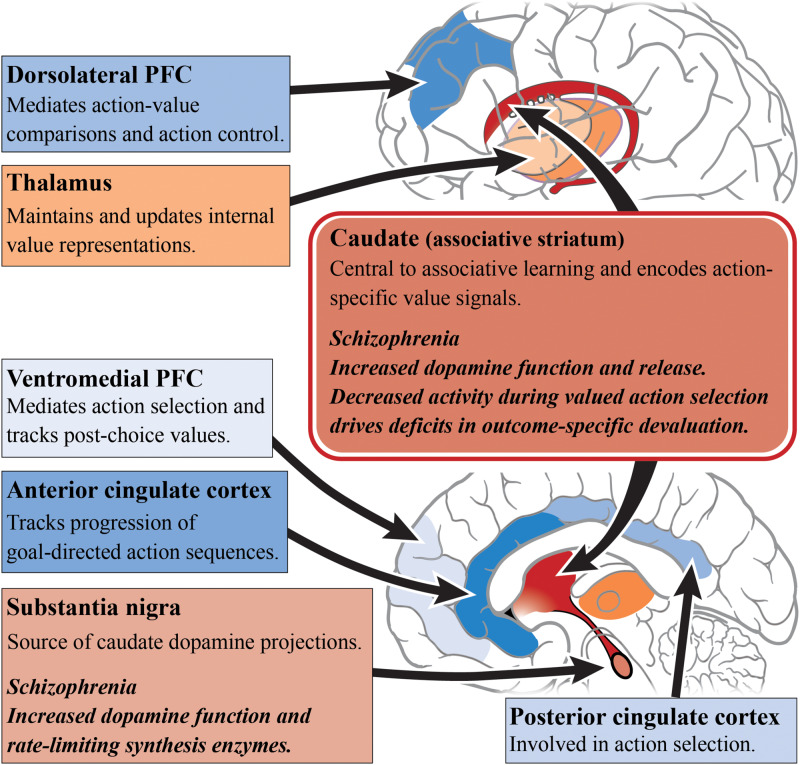
Goal-directed action and schizophrenia. A simplified diagram of the circuitry, subcortical (reds) and cortical regions (blues), and their roles in goal-directed action. Impaired dopamine function and release in the caudate/associative striatum (dark red) of patients living with schizophrenia may be the cause of impairments in goal-directed behavior. Increased dopamine function in the associative striatum may directly alter associative learning and the understanding of action-specific values. Alternatively, increased dopamine function may impair the integration of incoming cortical inputs. In particular, subregions of the prefrontal cortex have differing roles in the encoding of outcome values. Other cortical regions such as the anterior cingulate cortex and posterior cingulate cortex have also shown to have differing roles in action selection. *PFC*, prefrontal cortex.

When examining the neural substrates of action-outcome contingency learning, a number of studies have pointed to the role of the medial PFC and caudate, as activity in these regions varies based on the probability of an action being followed by an outcome (Tanaka et al., 2008; Liljeholm et al., 2011). Furthermore, subregions of the PFC appear to have specific roles in encoding the value of outcomes. For example, the dorsolateral PFC has been shown to mediate action-value comparisons and modulate action control (Morris et al., 2014), whereas, the ventromedial PFC is important for tracking post-choice values in order to update action values accordingly (Valentin et al., 2007; Tanaka et al., 2008; Morris et al., 2014). It has been suggested that connections between the dorsolateral PFC, OFC and caudate work as a circuit to compare action values for selection and, once a choice is made, update the action values (Morris et al., 2014). Another frontal cortical region implicated in goal-directed action is the ACC, with activity in this region reflecting the use of reward-type information to guide action selection (Noonan et al., 2011). This conclusion is supported by computational modeling, as the ACC has also been identified as being responsible for tracking the progression of goal-directed action sequences (Holroyd and Yeung, 2012; Shahnazian and Holroyd, 2018). This has direct implications for schizophrenia where there is abnormal functional connectivity with multiple brain regions, in particular the caudate and putamen (Yan et al., 2012), as seen in Figure 1. The role of the thalamus in subcortical integration has also been argued to be a key mechanism for maintaining and updating internal representations (Wolff and Vann, 2019).

In schizophrenia, caudate function appears to be central to deficits in goal-directed action. The outcome-specific devaluation task allows for the separate assessment of limbic and associative striatal involvement in decision-making, and is specific to goal-directed action because habitual behavior is resistant to outcome devaluation (Rossi and Yin, 2012). Using this task, it has been found that people with schizophrenia are capable of understanding changes in the value of outcomes after devaluation, but are unable to update their action selections accordingly (Morris et al., 2015). These behavioral deficits are driven by a decrease in caudate activity during valued actions, but not with changes in medial PFC activity, compared with healthy subjects. In a follow-up study, a contingency degradation task was used to further elucidate whether this impairment exists alongside habit formation or an impairment in instrumental learning (Morris et al., 2018). In this modified task, one of the action-outcome contingencies was degraded by delivering the outcome in the absence of an action. Those with schizophrenia were able to learn the best action to obtain rewards, but after contingency degradation, patients were unable to determine the more causal action. This suggests a core impairment in the learning of action-outcome associations, whereby people with schizophrenia are unable to encode the causal consequence of an action. Therefore, this impairment in goal-directed action is not driven by habit formation or an inability for instrumental learning but rather by an associative learning impairment.

### Preclinical Evidence of a Role for Dopamine and the Associative Striatum in Goal-Directed Behavior

**FIGURE 2:**
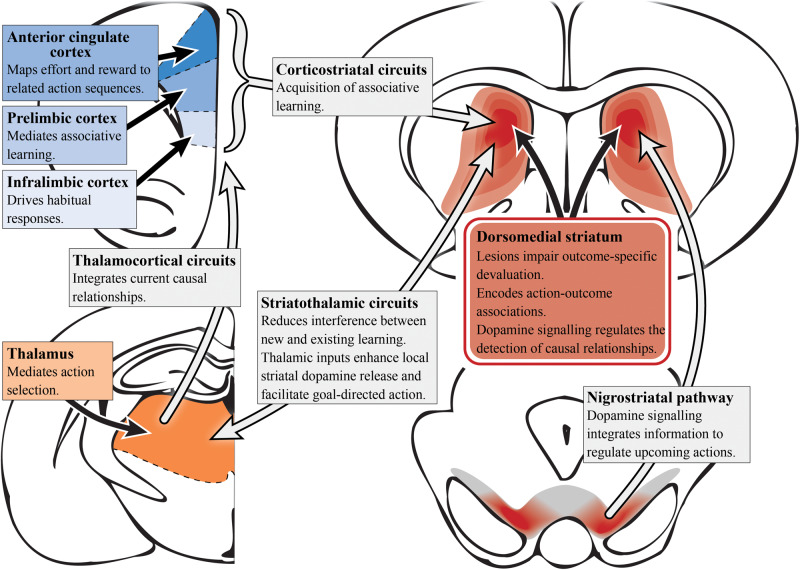
Goal-directed action and dopamine (preclinical studies). A simplified summary of the preclinical research on subcortical (reds) and cortical (blues) regions involved in goal-directed behavior with potential relevance to schizophrenia. Dopamine signaling driven by the nigrostriatal pathway projecting into the dorsomedial/associative striatum (dark red) is essential for associative learning. Aberrant functioning in the associative striatum could impact goal-directed behavior via multiple circuits. In particular, integrating and encoding inputs from cortical regions such as the anterior cingulate cortex, prelimbic and infralimbic cortices, which have distinct roles in terms of action selection, associative learning and habit formation. However, it is the corticostriatal circuit as a whole that is responsible for the acquisition of action-outcome associations. The thalamus also has an important role in mediating action selection with the thalamocortical circuit integrating causal relationships. Striatothalamic circuitry is important to managing learning in goal-directed behavior and also has a role in regulating striatal dopamine release.

A range of tools have been applied to manipulate the circuitry involved in goal-directed behavior in animal models (Rescorla, 1992; Johnson et al., 2005; Matamales et al., 2016). It is important for established operant tasks of relevance to schizophrenia to be used when assessing decision-making in rodents (Markou et al., 2013; Morris et al., 2015; Young and Markou, 2015; Der-Avakian et al., 2016). The neural basis of goal-directed action in rodents has been extensively examined, and suggests a complex convergence of multiple circuits that constitute the cortico-striatal thalamo-cortical feedback loop (Balleine et al., 2009), as illustrated in Figure 2. As described in schizophrenia patients, deficits in goal-directed action are seemingly driven by pathology in either the converging inputs to the associative striatum or their encoding within this region. Given that the associative striatum is the entry point for the basal ganglia, it is clear that this region has a highly regulatory role in action selection, planning and decision-making (Balleine and O’Doherty, 2010).

In rats, two components of cortico-striatal circuitry have been identified as being critical for goal-directed learning, the prelimbic cortex and the dorsomedial striatum (associative) which receives its input from the former region (Groenewegen et al., 1990). Using either outcome devaluation or contingency degradation, it has been shown that lesions of either of the aforementioned regions in rats impair the acquisition of associative learning, causing deficits in goal-directed action (Balleine and Dickinson, 1998; Corbit and Balleine, 2003; Yin et al., 2005b). Bilaterally disconnecting the prelimbic to associative striatal pathway in rats was shown to disrupt the acquisition of goal-directed actions, further supporting the functional roles of these regions in a corticostriatal circuit to mediate goal-directed behavior (Hart et al., 2018). Single-unit recordings in primates also have also demonstrated action-specific value signals in the dorsal striatum (associative), confirming the role of this region in the expression of goal-directed action as well as its aforementioned role in learning (Samejima et al., 2005; Lau and Glimcher, 2008). N-methyl-D-aspartate receptors in the posterior dorsomedial striatum (associative) are also important for encoding action-outcome associations during instrumental conditioning (Yin et al., 2005a).

The thalamostriatal pathway, linking the parafascicular thalamus with cholinergic interneurons in the posterior dorsomedial striatum (associative), is responsible for reducing interference between new and existing goal-directed learning (Bradfield et al., 2013). Moreover, the thalamocortical pathway is responsible for integrating current causal relationships (Alcaraz et al., 2018). Therefore, the preclinical evidence implicating the dorsomedial striatum (associative), and in particular the posterior portion, in goal-directed action supports the findings in humans suggesting a role for the caudate (associative striatum) in encoding action-outcome associations and establishing causal relationships (Balleine and O’Doherty, 2010). The infralimbic cortex has also been implicated in goal-directed action. Infralimbic inactivation in rats exhibiting habitual behavior (i.e., overtrained rats) saw reinstatement of sensitivity to outcome devaluation, suggesting heightened activity may impair goal-directed behavior (Coutureau and Killcross, 2003). In addition, neurons in the ACC have been shown to map anticipated effort and reward to their associated action sequences, further supporting the aforementioned studies in humans (Cowen et al., 2012).

In the context of dopamine systems, subcortical dopamine appears more relevant than cortical dopamine in the devaluation task. For example, dopamine function in the PFC is not necessary for the acquisition of instrumental learning, and although animals with dopaminergic lesions of the prelimbic cortex fail to adapt their actions to changes in contingency, their responses remain sensitive to outcome devaluation (Naneix et al., 2009). Moreover, dopamine depletion of the prelimbic cortex modulates the instrumental lever pressing rate but does not have a role in instrumental conditioning *per se* (Lex and Hauber, 2010). In contrast, studies on dorsomedial striatum (associative) dopamine signaling have shown no role in instrumental lever pressing but instead, the detection of causal relationships between an action and its outcome, i.e., associative learning (Lex and Hauber, 2010). It has also been demonstrated that the glutamatergic projections from the thalamus to the dorsal striatum (associative), activate striatal cholinergic interneurons to enhance local striatal dopamine release and improve goal-directed behavior (Cover et al., 2019). Stimulation of the substantia nigra induces striatal long-term potentiation and may positively reinforce the learning of behavior via dopamine D1 receptor-dependent potentiation of cortical inputs to the striatum (Reynolds et al., 2001; Wickens et al., 2007). Nigrostriatal dopamine signaling seemingly integrates diverse information required for the regulation of upcoming actions, as changes in the firing rate of nigrostriatal dopamine neurons, as well as dopamine signaling in the dorsal striatum (associative), have been found to accompany action selection (Howard et al., 2017). This dopaminergic signaling profile was found to be specific to behavioral choice and didn’t reflect reward prediction error, timing or value as single factors alone (Howard et al., 2017).

The role of dopamine in the dorsomedial striatum (associative) elucidated in these preclinical studies converges with the outcomes observed in schizophrenia, i.e., impaired associative learning and an inability to encode the causal consequences of their actions (Morris et al., 2018). This highlights the associative striatum as a prime target underlying impaired cognitive function in schizophrenia (Griffiths et al., 2014). This could in turn facilitate, or act in addition to, the corticostriatal dysconnectivity observed in schizophrenia, including reduced connectivity between the putamen and the medial PFC (Karcher et al., 2019), and large-scale disturbances in thalamo-cortical connectivity (Anticevic et al., 2014). Importantly, the available translational devaluation task provides a direct avenue to dissect the role of specific circuitry in preclinical models and explore targets that may rescue cognitive performance.

### Cognitive Flexibility in Schizophrenia

Decision-making behavior can also be controlled dynamically; a response or action can be selected when the outcome is desired, and equally, it can be withheld when the outcome is unwanted (Furlong and Corbit, 2018). This process is known as cognitive flexibility, an executive function that is underpinned by characteristics such as the formation of/shifting between attentional sets, response inhibition, perseveration and reversal of stimulus-response or action-outcome associations (i.e., reversal learning). Since cognitive flexibility is made up of several component processes, it has been shown that these differing forms of cognitive flexibility are governed by divergent forms of underlying neurocircuitry (Eslinger and Grattan, 1993). In humans and animal models, attentional set-shifting depends largely on the role of the medial PFC and ACC, as these regions are critical for flexibly shifting from one strategy to another (Birrell and Brown, 2000; Bissonette et al., 2013; Heisler et al., 2015). Response inhibition requires the recruitment of the dorsolateral PFC, ventrolateral PFC, ACC and the parietal cortex (Blasi et al., 2006; Hardung et al., 2017). It has also been shown that dorsal striatal D_2_-like receptor function mediates response inhibition in corticostriatal neural circuitry in humans (Ghahremani et al., 2012). Poor performance on an attentional set-shifting task has been observed in patients with schizophrenia due to a failure of inhibitory control and/or perseverative errors (Morice, 1990). Attentional set-shifting is also dependent on working memory, another cognitive process that relies on cortical function and is impaired in schizophrenia (Pantelis et al., 2009).

In contrast, reversal learning appears to be particularly sensitive to associative striatal function (Ragozzino, 2007; Braun and Hauber, 2011). However, as seen in studies in human and non-human primates, rules or strategies adopted during reversal learning may eventually dominate a response, advance too quickly and stifle learning assessments (Murray and Gaffan, 2006). As a result, reversal learning is primarily assessed using a probabilistic reversal learning task, which is used to reduce the ability to operate a basic strategy and to force the participant to apply accumulated evidence of previous actions and outcomes to guide choice (Hampton et al., 2007; Walton et al., 2010). This task examines flexible decision-making in the face of misleading feedback and the ability to rapidly shift responses based on positive or negative feedback (the increase or decrease in the likelihood of receiving a reward) when reward contingencies are reversed (Cools et al., 2002).

**FIGURE 3:**
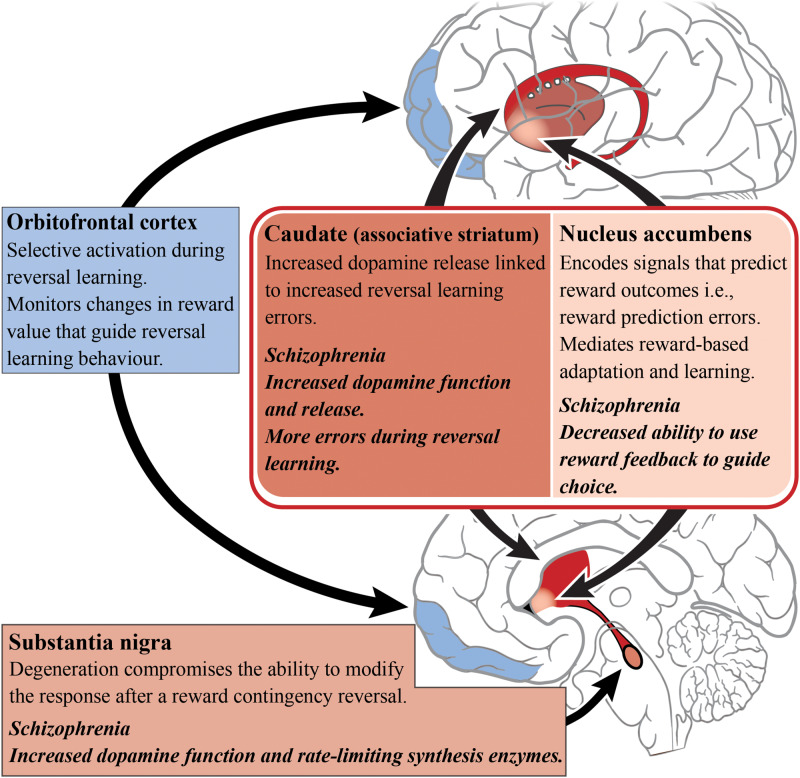
Reversal learning and schizophrenia. A simplified diagram of the circuitry, subcortical (reds) and cortical regions (blues), and their roles in reversal learning. Impaired dopamine function and release in the caudate/associative striatum (dark red) of patients living with schizophrenia may be the cause of reversal learning impairments. The dopamine enriched substantia nigra is involved in modifying responding to changes in reward contingencies and dopamine release in the caudate is related to reversal learning errors. In contrast, the nucleus accumbens has a role in predicting reward outcome. The orbitofrontal cortex is responsible for monitoring changes in reward value that guide reversal learning behavior.

The striatum has been implicated in reversal learning based on a number of functional imaging studies of reversal learning, with recruitment of both the ventral (limbic) and dorsal (associative) striatum being observed, as shown in Figure 3 (Rogers et al., 2000; Cools et al., 2002; Clarke et al., 2008; Tanaka et al., 2008). In the caudate (associative) specifically, dopamine receptor availability after methylphenidate administration accompanied drug-induced changes in reversal learning performance, i.e., larger increases in dopamine release corresponded with more reversal learning errors (Clatworthy et al., 2009). This is vital to our understanding of reversal learning impairments in schizophrenia as increased dopamine neurotransmission from the substantia nigra to the associative striatum is now considered a hallmark of the disorder. The nigrostriatal dopaminergic system has also been implicated in reversal learning, given that patients with Parkinson’s disease (where the neuropathology of the disease involves the degeneration of dopamine cells in the substantia nigra) exhibit a compromised ability to adapt to the reward contingency reversal (Peterson et al., 2009).

A host of cortical subregions, including the lateral OFC, inferior frontal gyrus, the dorsomedial PFC, the dorsolateral PFC and the posterior parietal cortex, have also been implicated in aspects of reversal learning performance (O’Doherty et al., 2001; Cools et al., 2002; Glascher et al., 2009; Mitchell et al., 2009). The OFC is particularly important in reversal learning as increased activity has been observed while participants perform reversals (as opposed to during the initial discrimination) which indicates the OFC’s role in the reformation of established associations (Ghahremani et al., 2010). People with OFC lesions also exhibit reversal learning deficits, suggesting an inability to learn from reward feedback and thereby indicating that the OFC is important for monitoring changes in reward value to guide behavior (Hornak et al., 2004).

A number of studies focusing on reversal learning have reported that limbic striatal dysfunction is tightly linked with specific reinforcement-driven reversal learning deficits observed in schizophrenia, most likely due to the interference with reward prediction error processing (Schlagenhauf et al., 2014). Some studies suggests that there are preliminary results in schizophrenia patients showing abnormal prediction error signaling, however, these findings remain inconsistent (Ermakova et al., 2018). Those with schizophrenia are able to acquire the initial probabilistic contingencies but achieve significantly fewer reversals than healthy matched controls, suggesting that OFC dysfunction is a prevalent aspect of the pathophysiology (Waltz and Gold, 2007). Therefore, there is a deficit in the ability to use this feedback and the prediction of reward outcome, in order to update internal reward value representations and guide choice (Waltz and Gold, 2007; Reddy et al., 2016). Interestingly, in a study examining probabilistic learning alone, no differences in limbic striatal reward-prediction-error activation were demonstrated between medicated patients and healthy controls, indicating that deficits in probabilistic learning in the disorder, may instead stem from processes outside of the limbic striatum (Culbreth et al., 2016b).

In a version of a probabilistic reversal learning task, schizophrenia patients achieved significantly fewer reversals than healthy controls and also showed a decrease in Win-Stay/Lose-Shift decision-making behavior (i.e., a decrease in the use of “winning” strategies) (Culbreth et al., 2016a). Furthermore, this behavioral deficit was linked with reduced activation (in comparison to controls) in striatal regions, and brain regions associated with cognitive control (Culbreth et al., 2016a). Studies in people experiencing first-episode psychosis have shown that there are both reinforcement and reversal learning deficits (Murray et al., 2008). These deficits in reversal learning are observed even when discrimination learning and attentional set-shifting remained intact, suggesting reversal learning may be a promising target for translational studies in early-stage schizophrenia (Leeson et al., 2009; McKirdy et al., 2009).

### Preclinical Evidence Dissecting the Circuitry Involved in Reversal Learning

**FIGURE 4:**
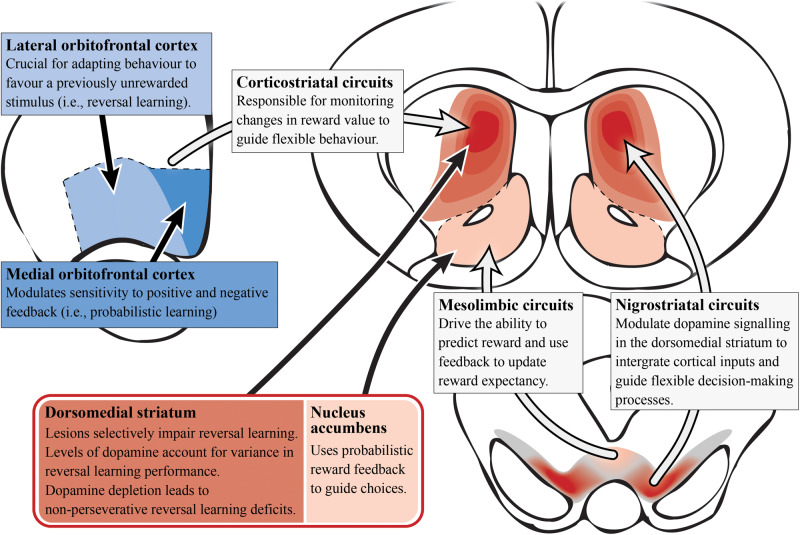
Reversal learning and dopamine (preclinical studies). A simplified summary of the preclinical research on subcortical (reds) and cortical (blues) regions involved in reversal learning with potential relevance to schizophrenia. Alterations in dopamine signaling in the dorsomedial/associative striatum (dark red; which is essential for reversal learning) could impair integration and encoding of inputs from other regions/circuits involved in probabilistic reversal learning behavior. This is most likely driven by the nigrostriatal circuit that modulates dopamine signaling in the striatum. In contrast, the nucleus accumbens is important for using probabilisitic reward feedback to guide choices (i.e., probabilistic learning). Corticostriatal circuitry monitors changes in reward value to guide choices. Specifically, the lateral orbitofrontal cortex allows the adaptation of behavior for reversal learning while the medial orbitofrontal cortex modulates reward feedback sensitivity for probabilistic learning.

Development of a translational task to examine probabilistic reversal learning in rodents has emerged in recent years, allowing researchers to probe the underlying neural circuitry involved (Bari et al., 2010; Ineichen et al., 2012; Dalton et al., 2016), as seen in Figure 4. Preclinical evidence supports a role for the associative striatum in action selection and for the OFC as an important cortical area for transforming affective feedback to behavioral adjustment (Xue et al., 2013; Izquierdo et al., 2017). Lesions of the dorsomedial striatum (associative) have been shown to impair a range of reversal learning paradigms in animals highlighting its complex role in managing cortical inputs to select and maintain particular computational strategies. For example, dorsomedial striatum (associative) lesions in monkeys produce a reversal learning phenotype similar to that observed after OFC lesions (Clarke et al., 2008; Castane et al., 2010), suggesting that the integration of OFC inputs can be selectively perturbed in the associative striatum. Lesions of the dorsomedial striatum (associative) in rats do not effect initial discrimination learning (Featherstone and McDonald, 2004; Ragozzino, 2007) but appear to affect the maintenance and execution of a selected strategy after a reversal (Ragozzino, 2007). Moreover, these lesions do not impact effort-related reward processes (Braun and Hauber, 2011), suggesting a specific role of the associative striatum in the computation of the reversal learning strategy rather than in the motivation toward a goal.

In contrast to the associative striatum, the role of the limbic striatum is more contentious. Lesions of the nucleus accumbens (limbic) in non-human primates disrupt spatial reversal learning but has no effect with visual cues, while in rats similar lesions have been shown to impair probabilistic reversal learning as they impact on the ability to use probabilistic reward feedback to guide action selection (Stern and Passingham, 1995; Dalton et al., 2014). However, based on a number of animal studies, there is also evidence of unaffected reversal learning following lesions to the nucleus accumbens (limbic), where dopamine dynamics are responsible for reward prediction errors (Burk and Mair, 2001; Schoenbaum and Setlow, 2003; Castane et al., 2010).

In rodent preclinical experiments, lesions of the OFC have also induced reversal learning deficits, while infralimbic and prelimbic cortical lesions (subregions of the rodent medial PFC) did not affect this process (Boulougouris et al., 2007; Ragozzino, 2007). Furthermore, the medial OFC modulates sensitivity to positive and negative feedback (indicating its importance for probabilistic learning), while the lateral OFC is crucial for adapting behavior to favor a previously unrewarded stimulus (important for reversal learning; Dalton et al., 2016). Interestingly, inactivation of the rat prelimbic and infralimbic cortices showed impairments in extradimensional task-switching, indicating that these medial PFC subregions may only be engaged in other forms of cognitive flexibility, and not in reversal learning specifically (Ragozzino et al., 2003). Most evidence suggests the medial PFC is only recruited for tasks involving a higher attentional demand and performance monitoring that require a shift in the strategy or rule (rather than the contingency) required to complete a task (Laubach et al., 2015). Seemingly, the OFC represents expected outcomes during reversal learning, possibly by utilizing value information stored in the region and/or deriving outcome information from subcortical networks tracking the reward environment (Cai and Padoa-Schioppa, 2014; Wassum and Izquierdo, 2015). The OFC projects to both the limbic and associative striatum, receiving reciprocal input via the mediodorsal nucleus of the thalamus, suggesting either area could work in concert with the OFC to direct reversal learning (Middleton and Strick, 1996; Schilman et al., 2008).

Studies in non-human primates have revealed that the striatum and OFC primarily modulate reversal learning via dopamine and serotonin signaling, respectively (Groman et al., 2013). Depleting dopamine in the OFC of non-human primates had no effect on reversal learning, whereas depleting dopamine in the striatum led to a non-perseverative reversal learning deficit (Clarke et al., 2007, 2011). In contrast, reducing serotonin signaling in the OFC impairs reversal performance by increasing perseveration (Clarke et al., 2004). Perseveration is the repetition of a behavior that occurs in the absence or cessation of a stimulus. So non-perseverative reversal learning deficits indicate that dopamine signaling in the associative striatum is not critical for the immediate adjustment to a reversal, but rather the subsequent acquisition and maintenance of a selected strategy in response to a reversal. It has been suggested that an optimal balance of dopamine D2 receptor function is required for ideal reversal learning performance (Izquierdo et al., 2012). This is supported in studies across mice, monkeys and humans that show low dopamine D2 receptor availability correlates with poorer reversal learning performance (Jocham et al., 2009; Groman et al., 2011; Laughlin et al., 2011). Lesions of the dorsomedial striatum (associative) also impair serial reversal learning but do not effect initial discrimination learning (Featherstone and McDonald, 2005; Ragozzino, 2007). This suggests that examining the serial reversal learning deficits in schizophrenia specifically (Brunelin et al., 2013), may allow us to better understand dopaminergic alterations in the associative striatum.

### Does Increased Associative Striatal Dopamine Function Compromise Cortico-Striato-Thalamic Circuits in Schizophrenia?

**FIGURE 5:**
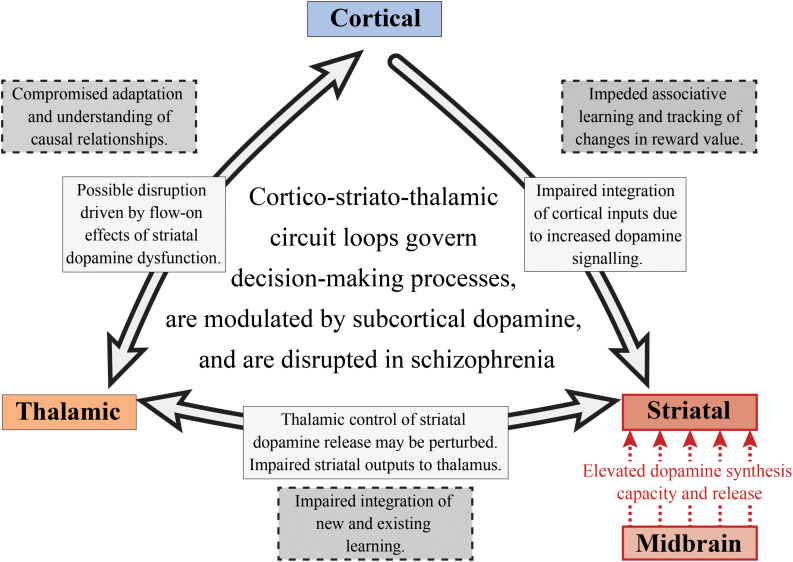
Subcortical dopamine, cognition and schizophrenia. This is a simplified diagram of the cortico-striato-thalamic circuit loop that is disrupted in schizophrenia. Increased striatal dopamine signaling, as well as the impaired integration of cortical inputs into the striatum, may affect a number of cognitive components involved in decision-making including those linked with goal-directed and flexible behavior. The nigrostriatal pathway (red) is responsible for the increased dopamine synthesis and release in the associative striatum. This could result in a perturbation of the thalamostriatal pathway, impacting striatal dopamine release and impairing the integration of new and existing learning. The corticostriatal pathway is also affected in schizophrenia as there is compromised integration of cortical inputs into the striatum, potentially impacting on associative learning and value tracking processes. Finally, this may have flow on effects for the thalamocortical pathway which would result in an inability to understand the consequences of actions and to appropriately adapt behavior.

It has been hypothesized that perturbations in cortico-striato-thalamic circuits play a major role in the pathogenesis of psychosis, which may also have implications for the global cognitive deficit observed in the disorder as well (Dandash et al., 2017). This hypothesis and its link with psychosis is often implied in the pathophysiological models of the disorder as the activity of these circuit loops are heavily modulated by dopamine (Robbins, 1990; Pantelis et al., 1992). As described in Figure 5, these loops generally act in a way that relays information from the cortex, through the basal ganglia, thalamus and then back to the cortex (Alexander et al., 1986). These circuits can act both independently and inter-dependently, whereby inputs from one loop can modify the output of other loops, allowing for the flexible modulation of internally generated and externally aroused behavioral responses to the environment (Haber, 2003). Based on information examining the specific neural circuits that mediate dopamine dysregulation, the circuit loop of greatest interest to schizophrenia research in cognition should be the dorsal “associative” loop. This loop relays information from the cortex to the associative striatum, then onto the pallidum and substantia nigra, and then finally onto the mediodorsal and ventral anterior nucleus of the thalamus, that then relays the information back to the cortex (Dandash et al., 2017).

Elevated dopamine function in schizophrenia is observed in both the substantia nigra dopamine cell bodies and their associative striatal terminals. Thus, altered dopamine transmission may be one of the fundamental mechanisms driving the disruption of the cortico-striato-thalamic circuit involved in decision-making (see Figure 5). Given that pathology in one part of a circuit rarely remains isolated, this will also affect the functions of interconnected systems (Fornito et al., 2015). Therefore, if we choose to examine cognitive processes that are selective for the associative striatum, such as goal-directed action and serial reversal learning, we will not only be able to understand the cognitive effects of subcortical dopamine alterations in schizophrenia, we will also be able to examine the effects on other components of cortico-striato-thalamic circuit loops. We suggest that in schizophrenia, impairments in goal-directed behavior and serial reversal learning may be due to perturbations in multiple components of the cortico-striatal-thalamic circuit loop. These disruptions may be driven by elevated dopamine synthesis and release from the midbrain into the associative striatum, which can hinder the maintenance and execution of decision-making processes. Impaired integration of cortical inputs into the striatum as a consequence of altered dopamine signaling may also be observed. This dysfunctional cortico-striatal pathway may then lead to impeded associative learning and an inability to track changes in reward value. For the thalamostriatal component of this circuit, thalamic control of striatal dopamine release may be disturbed as well as striatal outputs to the thalamus, impairing the integration of new and existing learning.

## Conclusion

Altered decision-making processes lead to inappropriate choices that further disadvantage people with schizophrenia through functional impairments and reduced quality of life (Kiwanuka et al., 2014). Antipsychotic medication is not effective in ameliorating these cognitive symptoms and there are currently no approved treatments, highlighting the need for novel investigative approaches (Kasper and Resinger, 2003; Lally and MacCabe, 2015). Emerging evidence suggests that dysfunction in the associative striatum, be it dopamine or otherwise, could precipitate the cognitive phenotypes observed in schizophrenia. This could occur due to direct changes in the associative striatal outputs or by impairing the integration of cortical inputs during decision-making. The complexity of these circuit loops, and decision-making processes in general, emphasizes that further research is required if we are to gain a better understanding of the underlying neurobiology of schizophrenia. We contend that research should now shift focus toward a better understanding of the role of specific striatal pathways in cognition, using tools that allow researchers to discretely manipulate circuitry in animal models and examine the effects through outcomes measured on sensitive cognitive tasks. For example, examining the role of the dopaminergic nigrostriatal pathway on goal-directed action could help us better understand the cognitive consequences of the increased dopamine function in the associative striatum observed in schizophrenia. In contrast, as serial reversal learning is relatively selective for cortico-striatal function, probing this process in animals could allow us to better understand the effects that altered associative striatal connectivity and circuit dynamics have on cognition in schizophrenia. For this reason, a detailed evaluation of the consequences of increased associative striatal dopamine function on cortico-striatal-thalamic circuitry and decision-making processes in preclinical models, is paramount.

## Author Contributions

K-AC, TB, and JK all contributed to the writing of the manuscript.

## Conflict of Interest

The authors declare that the research was conducted in the absence of any commercial or financial relationships that could be construed as a potential conflict of interest.
